# Chemistry-Based Modeling on Phenotype-Based Drug-Induced Liver Injury Annotation: From Public to Proprietary Data

**DOI:** 10.1021/acs.chemrestox.2c00378

**Published:** 2023-08-09

**Authors:** Mohammad Moein, Markus Heinonen, Natalie Mesens, Ronnie Chamanza, Chidozie Amuzie, Yvonne Will, Hugo Ceulemans, Samuel Kaski, Dorota Herman

**Affiliations:** † Department of Computer Science, Aalto University, Konemiehentie 2, 02150 Espoo, Finland; ‡ Predictive, Investigative and Translational Toxicology, PSTS, Janssen Research & Development, Pharmaceutical Companies of Johnson & Johnson, 2340 Beerse, Belgium; ∇ Pathology, PSTS, Janssen Research & Development, Pharmaceutical Companies of Johnson & Johnson, 2340 Beerse, Belgium; § Johnson & Johnson Innovation-JLABS, 661 University Avenue, CA014 ON Toronto, Canada; ∥ Predictive, Investigative and Translational Toxicology, PSTS, Janssen Research & Development, Pharmaceutical Companies of Johnson & Johnson, 3210 Merryfield Row, San Diego, California 92121, United States; ⊥ In-Silico Discovery, Janssen Pharmaceutica, Janssen Research & Development, Pharmaceutical Companies of Johnson & Johnson, 2340 Beerse, Belgium

## Abstract

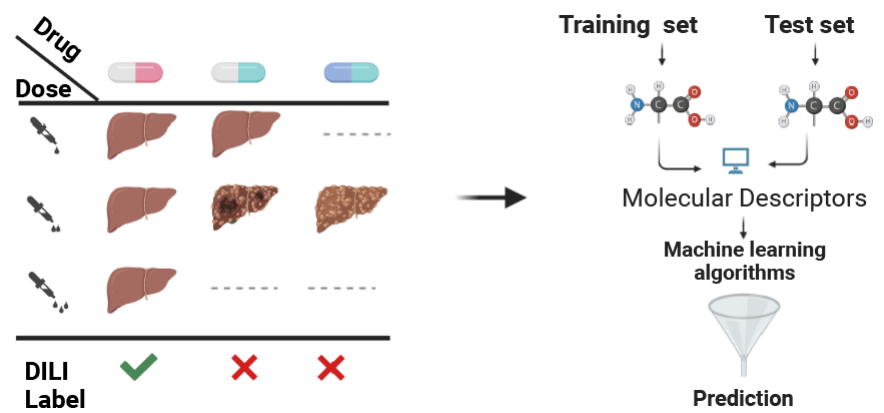

Drug-induced liver injury (DILI) is an important safety concern and a major reason to remove a drug from the market. Advancements in recent machine learning methods have led to a wide range of in silico models for DILI predictive methods based on molecule chemical structures (fingerprints). Existing publicly available DILI data sets used for model building are based on the interpretation of drug labels or patient case reports, resulting in a typical binary clinical DILI annotation. We developed a novel phenotype-based annotation to process hepatotoxicity information extracted from repeated dose in vivo preclinical toxicology studies using INHAND annotation to provide a more informative and reliable data set for machine learning algorithms. This work resulted in a data set of 430 unique compounds covering diverse liver pathology findings which were utilized to develop multiple DILI prediction models trained on the publicly available data (TG-GATEs) using the compound’s fingerprint. We demonstrate that the TG-GATEs compounds DILI labels can be predicted well and how the differences between TG-GATEs and the external test compounds (Johnson & Johnson) impact the model generalization performance.

## Introduction

Drug-induced liver injury (DILI) is a major problem affecting patients and pharmaceutical companies. DILI is the main reason for drug disapproval or market withdrawal.^1−3^ Therefore, developing a computational prediction model of DILI at an early stage of drug discovery will decrease the risk of failure in a drug development program.

There are no biomarker diagnoses that specify clinical DILI from other liver disorders with certainty and the diagnosis of DILI remains mainly based on ruling out other competitive causes.^4^ Regulatory agencies rely on drug postmarketing monitoring and clinical trials to discover adverse long-term effects.^5,6^ There are numerous public data sets^7−14^ from preclinical to postmarketing data capturing different aspects of drug toxicity information. These data sets often use agencies reports, drug labels, or scientific literature to annotate drug toxicity. In this way, information about the underlying mechanisms or dose dependency (dose dependent or intrinsic versus dose independent or idiosyncratic DILI) is lacking. The main limitation of those data sets is the generalization of hepatotoxicity, where hepatotoxicity is an umbrella term for several complex DILI phenotypes due to various mechanisms. We need to be able to differentiate primary from secondary, while we hypothesize the underlying mechanisms in addition to clinical risk factors.^4^ Furthermore, different data sets report conflicting DILI risks for similar drugs^15^ due to different criteria applied to case reports or drug labels which makes the reproducibility of them difficult.^8^

Few data sets collect toxicity information using subtle phenotypes based on controlled preclinical in vivo studies^16,17^ to assist early prediction of toxic drugs by in silico models. The Open Toxicogenomics Project-Genomics Assisted Toxicity Evaluation system^17^ (TG-GATEs) is a data set that includes toxicological data from both in vivo studies and in vitro assays in both rat and human hepatocytes. We extended this data set by performing a digital histopathology evaluation of liver slides that are publicly available on TG-GATEs data set. We carried out multiple phases to ensure that morphological diagnoses reflect the known underlying mechanisms of DILI in animals and translate the diagnoses to Standard for Exchange of Nonclinical Data (SEND) by applying International Harmonization of Nomenclature and Diagnostic Criteria (INHAND)^18^ compliant terms. This study collected data sets with a primary goal of generating accurate and reliable pathology data to be utilized as training data for machine learning (ML) algorithms while capturing primary pathology processes and, most likely, pathogenesis. We also used this annotation to classify TG-GATEs and proprietary Johnson & Johnson compounds into DILI-positive/negative groups for prediction model development and evaluation.

Several methods have been developed to predict DILI from molecular structure using predefined rules^19−26^ and ML based models.^27−31^ The latter reduces the task of predicting DILI label to a classification problem where the objective is to categorize each compound to DILI-positive/negative. A lot of studies^27,29,32,33^ developed many DILI prediction models using various shallow ML methods, including random forest (RF), decision tree, support vector machine (SVM), and logistic regression (LR) using various molecular structure representations such as extended connectivity fingerprint (ECFP).^34^ Chierici et al. employed deep learning to train multiple DILI prediction models using toxicogenomics data to compare their performance with shallow ML methods.^35^ They concluded that deep learning models do not systematically perform better than shallow ML methods yet due to the size of the data set or because their data set is not informative enough. Similarly, Kotsampasakou et al. demonstrated that data quality and preprocessing affect the performance significantly for shallow ML methods. Furthermore, Li et al. showed better performance of the ensemble of shallow ML methods (LR, RF, SVM and *k*-nearest neighbors) than deep models using Mold^2^ descriptor to predict DILI.^36^

This work describes a new DILI annotation approach solely based on in vivo studies and its prediction performance using ML models. We address previous works shortcomings by determining the compounds label (DILI-positive/negative) based on repeated dose-dependent in vivo assessments rather than drug labels or agency reports. Using compound chemical fingerprint, we assess the performance of a trained model using only TG-GATEs on two external test data sets, JNJ-I and JNJ-II. In this study, we focused on the prediction of preclinical study outcomes in industrial settings, where validation is both possible and feasible as a generalized model solely for preclinical data remains a complex task. This paper sheds new light on why the model fails to generalize to external test data sets. In particular, the model parameters and present chemical substructures influence model performance on the external test data sets. The main contributions of this work are as follows:

(1) Preparing and introducing a new DILI annotation that captures mechanistic and phenotypic DILI information more reliably and accurately than other data sets based on specific and standard in vivo liver histopathology nomenclature.
(2) Evaluating various ML methods for predicting DILI from compounds fingerprints.
(3) Studying the generalization of trained models from public data to proprietary pharmaceutical data sets.

## Methods

### Data Preprocessing

We collected observed phenotypes of TG-GATEs, JNJ-I, and JNJ-II compounds for different doses in the 29 day study. A compound was labeled DILI-positive if any phenotypes are observed regardless of the dose. For instance, those TG-GATEs compounds that showed any of the pathological end points defined in Table 6 are labeled DILI-positive and DILI-negative if nothing was observed. The Johnson & Johnson set was split into two: JNJ-I and JNJ-II ensuring a comparable number of compounds in each and a similar distribution of DILI-positive/negative.

### Compound Fingerprint

Compounds are represented by ECFP^34^ derived from their molecular structure. ECFPs are circular topological fingerprints designed to represent the presence or absence of a particular molecular substructure circular neighboring atoms. We used a diameter of 8 with a length of 4096.

### Embedding Analysis

Different dimensionality reduction algorithms were employed to project the data set to two-dimensional space to visually compare TG-GATEs and Johnson & Johnson compounds. In this study, PCA^37^ and UMAP^38^ are performed on ECFP fingerprints to visualize the compounds in two-dimensional space. We used scikit-learn^39^ and UMAP^40^ to compute the embeddings.

### Predictive Model Formulation

Let **x** = (*x*_1_, *x*_2_, ..., *x*_4096_) ∈{0, 1}^4096^ denote a binary ECFP fingerprint of size 4096 and *y* ∈ {0, 1} is the corresponding binary DILI label. Given a training set,  consisting of *N* compounds drawn from an unknown distribution. Let *N*_0_, *N*_1_ denote the number of DILI-positive and DILI-negative, respectively. The goal is to train a classifier to predict the DILI label of a compound by optimizing a certain objective function (i.e., cost or loss function). The distribution of DILI-positive/negative is uneven within our data set (Table 2). There are multiple approaches to alleviate this issue including data sampling and cost-sensitive learning.^41^ We used cost-sensitive learning with a balanced strategy where it gives higher weights to minority class and lower weight to majority class. Weights associated with classes are denoted by . In the standard learning setup, we considered *w*_0_ = *w*_1_ = 1; effectively no strategy was used to address *y*-skewed data distribution.

### Random Forest

RF^42^ is a supervised estimator that fits a number of decision trees induced from numerous subsamples of a data set. RF is a well-known classical ML algorithm in several drug toxicity prediction models.^28,29^ The goal is to optimize split function parameters over each node to send the arriving data point **x** to its left or right children until reaching a leaf node. The split function at node *j* to arrange the arriving data point, , to left or right children is determined by minimizing one of the following cost functions:
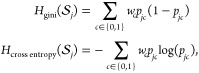
where *p*_*jc*_ denotes the empirical probability of label *c* computed on the arriving training *S*_*j*_. Each tree classifies an input compound by recursively branching out the data set to subgroups based on a single feature (fingerprint bit). Finally, it assigns the most probable label to each subgroup. The significant benefit of using RF is providing an importance score of each feature that shows each fingerprint bit’s contribution to the prediction task. RF shape and behavior are influenced by a few parameters:

- The number of decision trees, *T*.
- The maximum allowed tree depth, *D*.
- The choice of optimization objective function (split function), *H*.

### Support Vector Machine

SVM^43^ is a supervised estimator that finds the best decision boundary represented by a hyperplane parametrized by **θ** with the largest margin to assign toxicity class to the input compounds. The main idea behind the SVM algorithm is to transform the data to higher dimensional data using a suitable function, ϕ(*x*), where the classification task is simpler such that
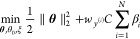
1subject to 
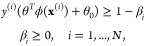
where *C* > 0 is the regularization coefficient. SVM behavior is influenced with

- The regularization coefficient *C*.
- The kernel function such as linear or RBF.

### *L*_1_-Logistic Regression

LR model is a supervised linear estimator that estimates the probability of a compound being toxic. *L*_1_-regularized LR is known to avoid overfitting and have a good generalization performance.^44^ In the logistic model, the probability of a compound **x** being toxic has the form:
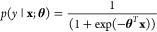
2where **θ** ∈ **R**^4096^ are the parameters. Under the Laplacian prior, given by *p*(θ) = (*C*/2)^4096^ exp(−*C* ∥ **θ**∥_1_), the maximum a posterior estimate of **θ** is
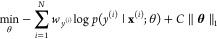
3where *C* is a regularization coefficient and ∥.∥_1_ is the *L*_1_ norm.

### Model Hyperparameter Grid Search

**Chart 1:**
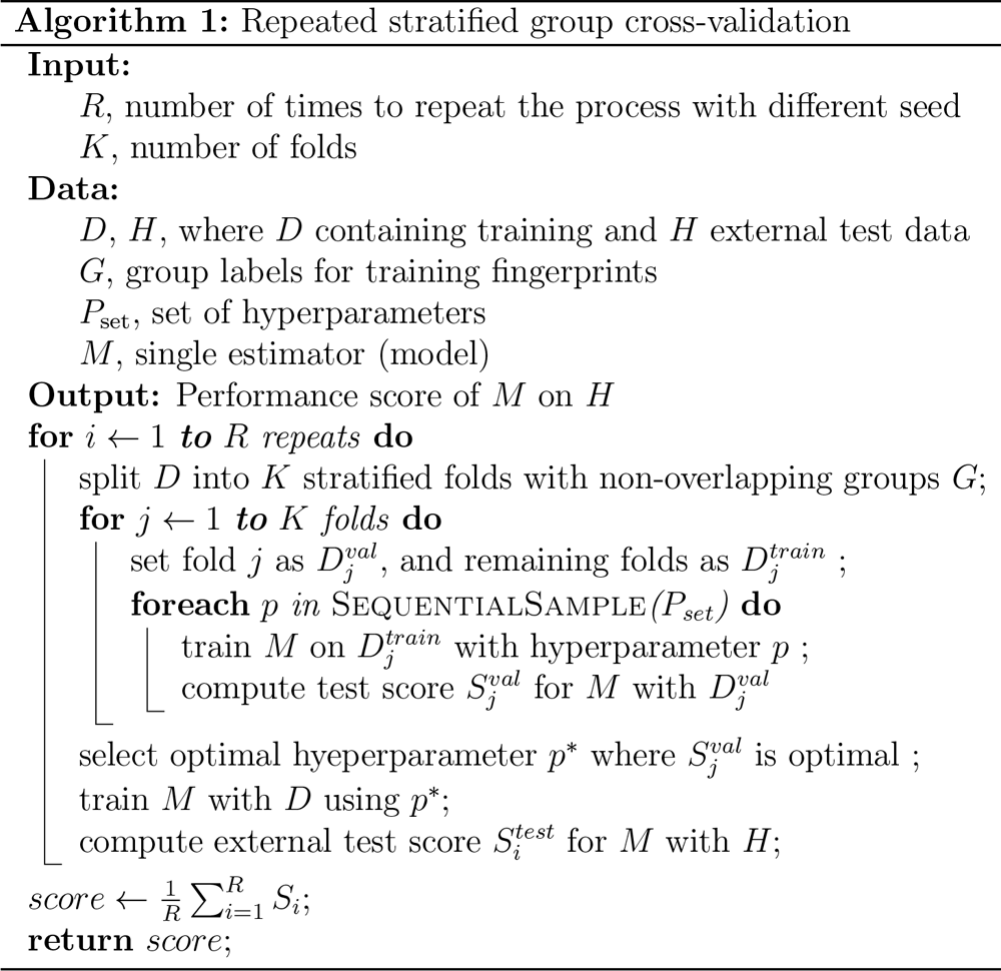
Algorithm 1

We used scikit-learn^39^ implementation to train RF, SVM, and *L*_1_-LR models. We performed a grid search over the model’s hyperparameter, as summarized in Table 1. Specific hyperparameters search space are evenly sampled over the defined interval in log space. For instance, parameter *T* for RF is sampled evenly in log space from 1 to 2^9^ which results in a search space of 1, 2, 4, 8, 16, 32, 64, 128, 256, 512 for the number of trees, *T*. The grid search follows the procedure explained in Chart 1.

**Table 1:** Hyperparameter Grid Search Values

Model	Parameter	Support	Sample count
RF	*T*	[1,2^9^]	10
		{1, 2, 3, 4, 5,	–
		6, 7, 8, 9,	
	*D*	10, 15, 20, 25,	
		30, 35, 40, 45,	
		50, 55, 60, 65,	
		70, 75, 80, pure}	
	*H*	{cross entropy,	
		gini}	–
SVM	*C*	[2^–5^, 2^15^]	10
	kernel	{linear, rbf}	–
	γ	[2^–15^, 2^3^]	10
*L*_1_-LR	*C*	[10^–4^, 10^4^]	100

### Training Procedure and Model Validation

Each model’s prediction performance and hyperparameter search is evaluated using a 5-fold stratified group cross-validation with 10 repeats as shown in Chart 1. We used the area under the receiver operating characteristic curve (AUROC) score^45^ to find the best hyperparameter. We report the performance of each model in predicting the DILI label on JNJ-I and JNJ-II using AUROC, precision (PR), recall (RE), specificity (SP), and Matthews correlation coefficient (MCC). The algorithm splits the training data (TG-GATEs) into stratified five-fold with a nonoverlapping group. The group labels are acquired by running Butina clustering algorithm^46^ using Tanimoto distance. We used such procedure to prevent predicting compounds with high group Tanimoto similarity to the training data producing more realistic scenario to assess the models prediction performance.^47^

## Results

We built multiple DILI prediction models trained on TG-GATEs,^17^ and their generalization performance is evaluated on JNJ-I and JNJ-II. We used the above annotation method in this study to process the data. The Methods section explains the details of the preprocessing steps and model definition.

### Data Set

#### Data Collection

Our main goal was to prepare a large data set accurately capturing DILI and the likely underlying mechanisms to be utilized eventually in ML methods. Therefore, we performed a digital histopathology evaluation of liver slides that are publicly available on TG-GATEs data set, a database containing scanned digital microscopic slides of the liver, generated from various experiments involving more than 100 compounds. Our review included the evaluation of slides from studies with a total of 83 hepatotoxicants carefully selected to reflect a wide range of pathology manifestations and possible mechanisms of DILI. Slides were generated at multiple time points: 4 h, 24 h, 4 days, 7 days, 15 days to 29 days in most of the studies. The histological evaluation involved 4 phases, which were as follows:

(1) Confirming or identifying new diagnoses and providing all primary morphological diagnoses present in the liver sections of all animals in the study and at every time point.
(2) Translating each of the diagnoses to SEND-compliant terms that align with INHAND nomenclature, using descriptive rather than interpretative diagnoses.
(3) Identifying possible primary pathology processes and differentiating them from adaptive or secondary changes, such as *ischemic necrosis of centrilobular hepatocytes* as a result of *hepatocyte hypertrophy*; or liver pathology findings considered in response to lesions occurring from outside the liver. Furthermore, spontaneously occurring background findings such as *inflammatory infiltrates* or *focal single cell necrosis* were excluded.
(4) Providing the most likely pathogenesis and possible adverse outcome pathway for the liver pathology findings by analyzing the chronological progression of the findings from the earliest time points to later time points.

Phases 1 and 2 involved careful consideration of the morphological diagnoses to ensure all relevant morphological changes were captured, and the diagnoses were streamlined to reflect primary pathologies that fit into the known mechanisms of DILI in animals. Besides translating the diagnoses to INHAND^18^ or SEND compliant terms, an attempt was made to harmonize nomenclature across studies by capturing the main morphological diagnoses without including cumbersome modifiers that are not amenable to merging with liver pathology findings from other studies. Phase 3 was critical in determining if the liver was indeed a target of the drug/chemical by analyzing all associated changes in the animals, in addition to compounds that might cause liver pathology findings secondary to other systemic changes, such as *Kupffer cell hyperplasia/activation* following NSAID compounds that produce intestinal ulceration and peritonitis. Therefore, this stage included analysis of all supporting data from clinical observations, clinical pathology and the pathology reports. Phase 4 involved identifying the earliest changes and the earliest time point at which they occurred, by looking for very subtle changes, capturing their zonal distribution and subanatomic localization, and mapping out the lesion progression and the likely pathogenesis of the lesions. Table 6 was complemented by a consultation of the literature on the known targets and mechanism of injury (wherever available) for the compound, in order to determine the pathogenesis and develop a likely adverse outcome pathway (AOP). We prepared three data sets, namely TG-GATEs, JNJ-I and JNJ-II, including both public and proprietary compounds. The proprietary compounds were divided into JNJ-I and JNJ-II with a similar distribution of positive/negative as shown in Table 2.

**Table 2:** Data Sets Used for Model Development and Evaluation

		DILI	
Name	*N*	negative	positive
TG-GATEs	117	44	73
JNJ-I	155	110	45
JNJ-II	158	110	48

Table 3 compares the overlapping in terms of DILI-label in common compounds between TG-GATEs, DILIrank^7^ and DILIst.^12^

**Table 3:** Proposed TG-GATEs DILI Label Overlapping on Common Compounds

		DILI	
		negative	positive
DILIrank	Ambiguous DILI	5	2
	Less-DILI	13	22
	Most-DILI	15	30
	No-DILI	2	3
DILIst	DILI Positive (1)	28	58
	DILI Negative (0)	5	4

#### Chemical Similarity

**Figure 1:**
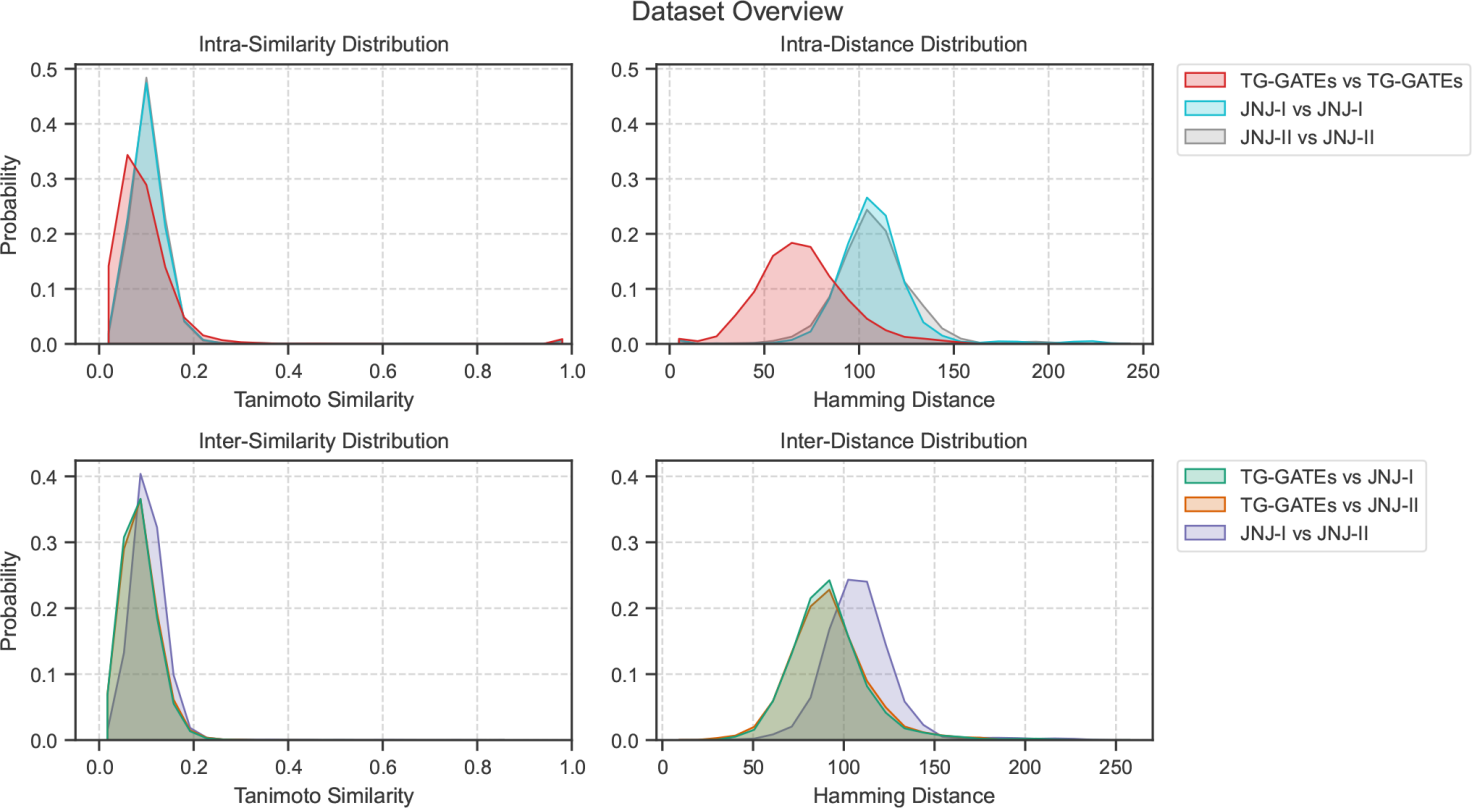
Compounds similarity and distance distribution showing that TG-GATEs data set populates different compounds that are not close to JNJ-I or JNJ-II.

**Figure 2:**
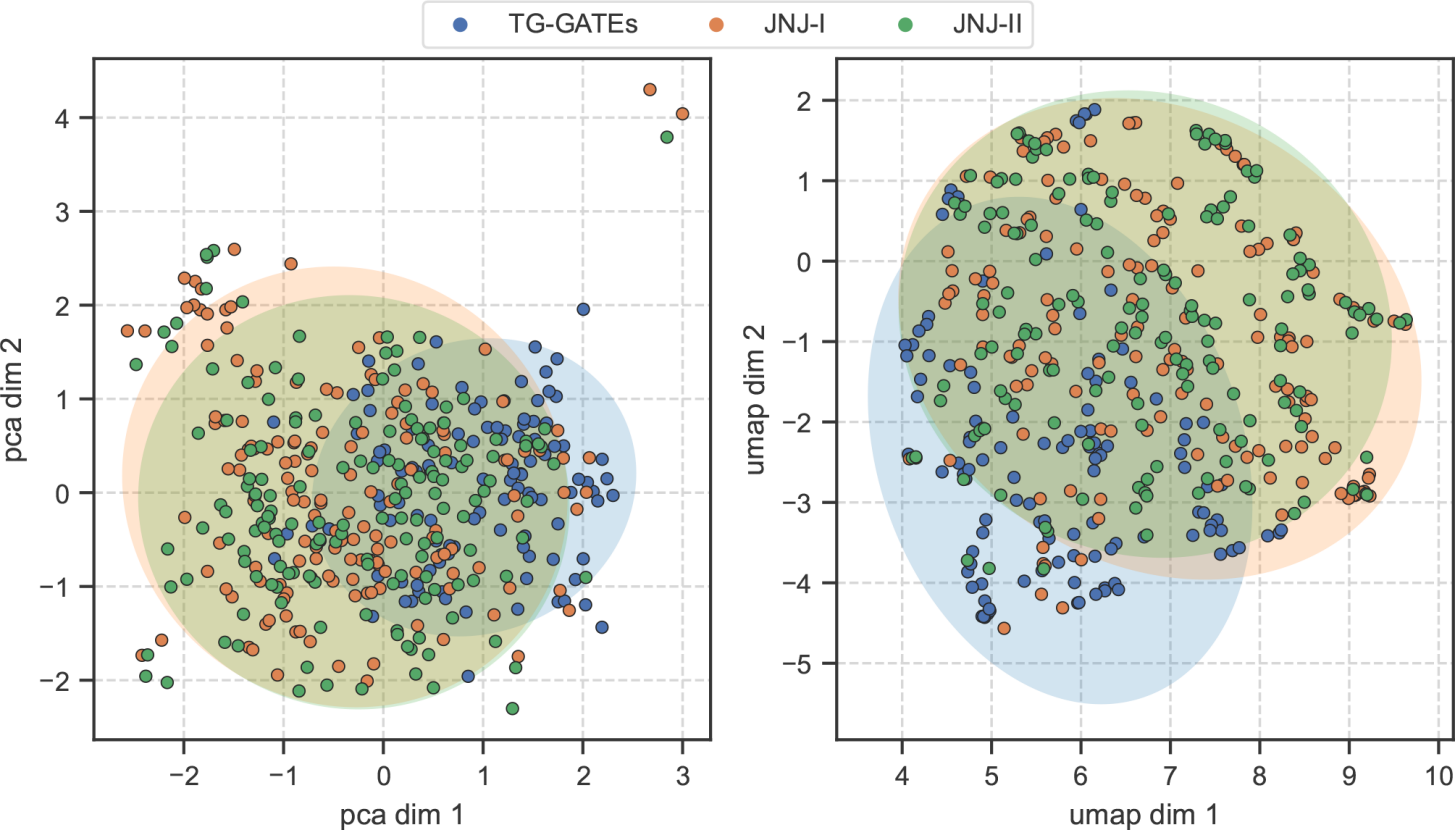
Scatter plot for TG-GATEs, JNJ-I and JNJ-II colored by their corresponding data set. This better shows how TG-GATEs compounds (blue dots) are located further away from JNJ-I and JNJ-II.

We computed the distribution of Tanimoto similarity and hamming distance between all pairs of compounds from TG-GATEs to JNJ-I and JNJ-II, as shown in Figure 1. This graph indicates that many compounds in TG-GATEs are not close to those in JNJ-I and JNJ-II. Furthermore, Figure 2 illustrates the projection of all the compounds to the same chemical embedding using PCA and UMAP. It indicates visually that many of the TG-GATEs compounds are covering a different chemical space than JNJ-I, or JNJ-II.

#### Predictive Modeling

We compared the performance of RF, SVM and *L*_1_-LR models trained on TG-GATEs using ECFP molecular fingerprints to predict compound DILI label. Models were trained within the framework of 5-fold group cross-validation with 10 repeats to decrease the effect of randomness on the prediction model performance. In addition, the generalization performance of the trained models was evaluated on JNJ-I and JNJ-II. We used the AUROC to measure the performance. A significant drop in generalization performance was observed for all models when tested against both JNJ-I and JNJ-II external sets. Tables 4 and 5 summarize the model’s prediction performance on the external and training data sets. RF and SVM models achieved higher AUROC score than *L*_1_-LR across 10 repeats, but all models performed roughly 14 % worse than their CV performance.

**Table 4:** Cross-Validation AUROC Performance Score

Train Set	TG-GATEs		TG-GATEs, JNJ-I	
Model	standard	cost-sensitive	standard	cost-sensitive
*L*_1_-LR	0.64 ± 0.05	0.64 ± 0.05	0.67 ± 0.03	0.67 ± 0.03
RF	0.68 ± 0.04	0.67 ± 0.04	0.64 ± 0.03	0.65 ± 0.03
SVM	0.69 ± 0.03	0.68 ± 0.03	0.67 ± 0.01	0.66 ± 0.01

**Table 5:** Performance Score on the External Test Sets

			JNJ-I		JNJ-II	
Train	Method	Metric	standard	cost-sensitive	standard	cost-sensitive
TG-GATEs	LR	AUROC	0.57 ± 0.02	0.57 ± 0.02	0.49 ± 0.02	0.48 ± 0.03
		MCC	0.10 ± 0.02	0.10 ± 0.06	–0.04 ± 0.05	–0.02 ± 0.07
		PR	0.32 ± 0.01	0.32 ± 0.01	0.29 ± 0.02	0.29 ± 0.02
		RE	0.72 ± 0.04	0.78 ± 0.11	0.64 ± 0.09	0.70 ± 0.13
		SP	0.39 ± 0.03	0.31 ± 0.07	0.32 ± 0.06	0.27 ± 0.09
	RF	AUROC	0.59 ± 0.01	0.60 ± 0.02	0.52 ± 0.03	0.56 ± 0.04
		MCC	0.12 ± 0.03	0.07 ± 0.04	0.06 ± 0.08	0.04 ± 0.05
		PR	0.31 ± 0.01	0.30 ± 0.01	0.32 ± 0.02	0.31 ± 0.01
		RE	0.92 ± 0.05	0.98 ± 0.02	0.91 ± 0.04	0.99 ± 0.01
		SP	0.18 ± 0.05	0.06 ± 0.04	0.14 ± 0.10	0.03 ± 0.03
	SVM	AUROC	0.57 ± 0.02	0.55 ± 0.05	0.51 ± 0.02	0.49 ± 0.02
		MCC	0.06 ± 0.10	0.05 ± 0.07	–0.02 ± 0.03	–0.01 ± 0.03
		PR	0.25 ± 0.13	0.31 ± 0.02	0.23 ± 0.12	0.30 ± 0.01
		RE	0.59 ± 0.41	0.94 ± 0.10	0.54 ± 0.40	0.90 ± 0.16
		SP	0.47 ± 0.37	0.12 ± 0.17	0.44 ± 0.39	0.09 ± 0.14
TG-GATEs, JNJ-I	LR	AUROC	–	–	0.49 ± 0.01	0.49 ± 0.01
		MCC	–	–	0.01 ± 0.01	0.00 ± 0.03
		PR	–	–	0.31 ± 0.01	0.30 ± 0.02
		RE	–	–	0.37 ± 0.01	0.33 ± 0.04
		SP	–	–	0.64 ± 0.01	0.67 ± 0.02
	RF	AUROC	–	–	0.50 ± 0.03	0.52 ± 0.02
		MCC	–	–	0.03 ± 0.02	0.03 ± 0.06
		PR	–	–	0.32 ± 0.01	0.33 ± 0.05
		RE	–	–	0.36 ± 0.08	0.26 ± 0.07
		SP	–	–	0.67 ± 0.08	0.77 ± 0.03
	SVM	AUROC	–	–	0.54 ± 0.02	0.54 ± 0.02
		MCC	–	–	0.10 ± 0.04	0.09 ± 0.02
		PR	–	–	0.41 ± 0.05	0.40 ± 0.03
		RE	–	–	0.24 ± 0.08	0.22 ± 0.06
		SP	–	–	0.84 ± 0.10	0.85 ± 0.06

#### Imbalanced Data Set

The DILI-positive/negative compounds ratio is not equal (Table 2), leading to an imbalanced data set due to which might affect the model performance. We addressed this problem using a cost-sensitive learning algorithm to train the models. Overall, using cost-sensitive learning slightly improved the RF model generalization performance, decreased the performance for SVM and had no effect on *L*_1_-LR.

#### Chemical Substructure Importance Weight

**Figure 3:**
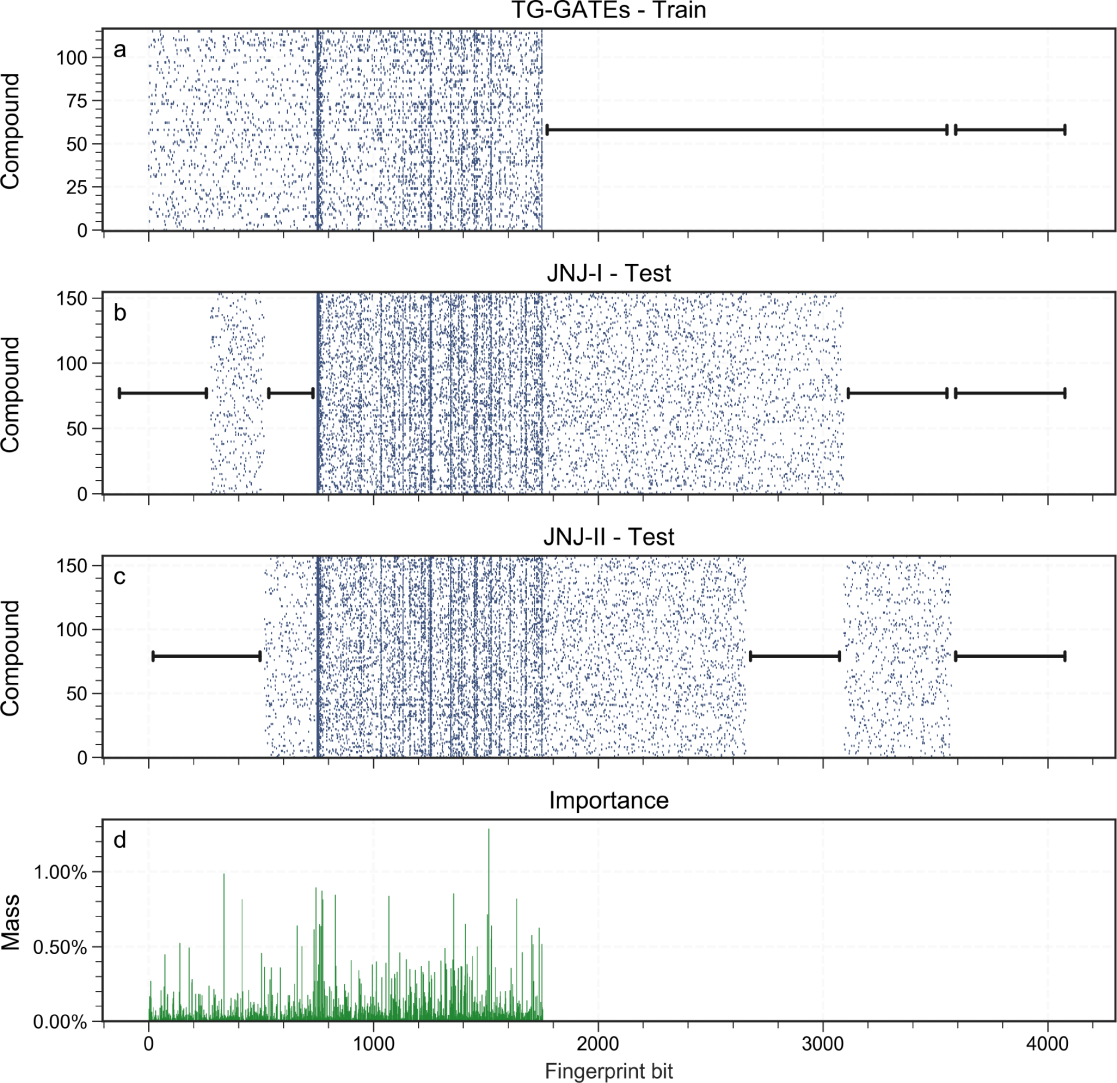
Random forest importance. Panels (a–c) show each data set compound’s fingerprint. There are multiple missing fingerprint bits in each data set. Therefore, RF model assigns a significant mass on those fingerprint bits missing in JNJ-I and JNJ-II as illustrated in panel (d), which leads to poor generalization performance.

We also examined the chemical substructure (fingerprint bit) average importance weight given by the RF model trained on TG-GATEs in Figure 3. This virtually shows the missing fingerprint bits between the training data and external test sets, leading RF model to ignore them by assigning a negligible importance. Essentially, the RF model ignores present chemical information in JNJ-I and JNJ-II when predicting the DILI label, which contributes to poor generalization.

## Discussion

Assessing DILI risk associated with drugs is difficult and complex,^48^ but it is necessary to accelerate the effort to evaluate and discover new DILI biomarkers. Chen et al. asserted that “there is no commonly adopted practice by which the research community can classify a drug’s DILI potential in humans”.

### Human DILI

The introduced annotation framework and corresponding data set have been developed with the primary goal of improving early DILI detection models in preclinical settings. The limited understanding and prediction of DILI is primarily due to the discrepancies between human and animal DILI observed during preclinical drug development and the complex mechanism of human DILI.^49,50^ These factors severely hinder preclinical efforts to accurately predict DILI and uncover the underlying in vivo mechanisms. Furthermore, existing in vitro hepatocyte systems based on human cell lines or rodent hepatocytes are not optimal when simulating human DILI.^51^ As a result, preclinical toxicity models lack universal predictability of drug efficacy in humans^52^ and are largely due to metabolic differences between species, resulting in setbacks within pharmaceuticals company developing new drugs.^53−58^ Although the detection of DILI in humans is crucial, we did not add any data enabling us to learn the translation from in vivo study to human DILI and focused on building early preclinical DILI model.

### Drug Labeling

There have been numerous attempts to build data sets where DILI risk is determined based on the existence of specific keywords on the drug label^7^ or case reports^13^ from approved agencies, literature, etc. Therefore, DILI labels are partially affected by the opinions and choices of the authors rather than evidence^8^ given the uncertainty of the causality assessment. Alternatively, we developed a new methodology to determine DILI labels based on particular in vivo studies. We could generate peer-reviewed, fit for purpose and INHAND-compliant pathology data for use in ML. Implementing INHANDS provides standardized terminology and diagnostic criteria for preclinical studies. It facilitates data integration and enhances the interpretation of pathological findings. Furthermore, it helps in unraveling the underlying mechanisms of pathological changes, thus enabling a deeper understanding of the physiological responses to drugs. Classic hepatotoxicants were associated with a variety of hepatobiliary pathology and changes in liver enzymes. Several pharmaceutical compounds that have been withdrawn from the market or have a black box warning for causing idiosyncratic DILI did not produce overt pathology in rats. However, in most of these studies, consistently cytoplasmic tinctorial or degenerative changes associated with glycogen depletion were present in the periportal areas. These are subtle changes that would not be typically recorded in a regular toxicological study since most of them were only transiently present between hours postdose and 4 days.

Histopathology of preclinical species might not be able to predict idiosyncratic and immune mediated DILI, but other tools, such as toxicogenomics might be helpful since some of the early underlying mechanisms might be similar between animals and human. The majority of primary lesions and clinical chemistry changes seen with classic toxicants were present by day 4, suggesting that screening compounds for hepatotoxicity can be achieved in a 4 day study. When primary lesions were recorded in order of frequency of induction by the reviewed compounds, the most commonly encountered liver pathology findings were *hepatocellular hypertrophy*, *periportal glycogen depletion*, *cytoplasmic vacuolation* (due to peroxisome proliferation, SER proliferation, accumulation of lipids, etc.), *apoptosis/single cell necrosis* as well as *zonal necrosis*, and *increased mitoses* (almost always associated with *hypertrophy* and transiently occurring and peaking at day 4). When generating pathology data for ML, recording early changes such as *increased mitoses* or *cytoplasmic alteration* is critical. These changes tend to be transient, but possibly part of the early key events in the pathogenesis of lesions observed at later time points. These early changes are also likely to correlate with toxicogenomic data if collected at similar early time points.

In some instances, the propsed drug labeling in this study does not align by DILIrank^7^ or DILIst^12^ as shown in Table 3. The absence of detail criteria taken by DILIrank/DILIist for assigning the label makes it difficult to reason for such discrepancies. It mighe be attributed to difference between human and rat metabolizim. Consequently, we were unable to compare our results with those data sets, for instance, based on our labeling drug hydroxyzine is DILI-positive but DILIrank assigns a label of No-DILI.

### Predictive Model

Independent of the models, we observed a key trend where the performance dropped significantly on the external test set, JNJ-I and JNJ-II, while achieving good cross-validation scores on TG-GATEs. We attribute the failure of generalization mainly due to the intrinsic difference between Johnson & Johnson compounds and TG-GATEs. Therefore, the external test data sets cover different regions of chemical space, while the model shows better performance for TG-GATEs, leading to an imperfect prediction model. For instance, we showed the RF model ignores many present chemical substructures in JNJ-I and JNJ-II compounds for predicting the DILI label, which subsequently affects the generalization performance.

The uncertainty in the DILI label could also contribute to the lack of generalization. Not all compounds fit into a particular pathology end point; on the contrary, many compounds satisfy the criteria for multiple end points in our case where we collected information for multiple doses. Training a separate model for each pathology end point was impossible due to the limited data (Table 6). Our DILI-positive/negative labeling approach to translate pathology readings effectively gathers all various pathology end points under one umbrella class of DILI-positives. Admittedly, this inherently makes it harder for a model to learn DILI-positive where representing several mechanisms.

## Conclusion

We introduced a phenotype-based DILI annotation that is more reliable, accurate, and reproducible in comparison with previous works despite poor generalization to the external data set. Characterization of DILI label by phenotypes makes it easier to hypothesize causality and eliminates the need to define opinion-based criteria to analyze the agencies report, drug labels, etc. As a result, it is easier to reproduce more compounds for monitoring to build a prediction model with better generalization. Three data sets were defined using the introduced annotation: TG-GATEs, JNJ-I, and JNJ-II. RF, SVM, and LR models trained on TG-GATEs achieved a good cross-validation score but failed to generalize to the external test data sets JNJ-I and JNJ-II. Our investigations so far have only been on a small scale of compounds; we have shown that the difference in the data distribution of the molecular substructure impacts model parameters. In particular, TG-GATEs and Johnson & Johnson data set cover non-overlapping regions of chemical space, causing the model to ignore information in a subset of the present Johnson & Johnson compound’s substructures.

We are currently studying to build a dose–response model for each phenotype to address the uncertainty in labeling approach. This will allow us to avoid mixing various mechanisms under the same label and predict phenotype findings instead of DILI-positive/negative. By removing the fixed time of 29 days in vivo studies, we aim to create a better model. Furthermore, we plan to explore the possibility of using in vitro assay to improve generalization performance. Our findings have shown that differences in molecular substructure distribution can affect the model’s performance, and increasing the size of the training set or augmenting it with side information such as in vitro assay may be a way to overcome this issue. Overall, while acknowledging the limitations and areas for improvement, our approach has the potential to enhance the accuracy and reliability of dose–response modeling, which can have important implications for drug development and safety evaluation.

## Appendix 1: Pathological End Points

The data were derived from 24 h to 29 days tox studies in the rat. Most primary liver pathology findings were observed from day 4 observations. We prepared our data sets based on 29 days readings, and the pathologic effects of TG-GATEs compounds on liver tissues are categorized into DILI phenotypes as listed in Table 6.

**Table 6:** TG-GATEs Observed Phenotype Counts

	Phenotype	*N*
1	apoptosis	2
2	apoptosis/single cell necrosis	1
3	atypia nuclear	1
4	bile duct hyperplasia	4
5	cytoplasmic alteration basophilic change/	6
	glycogen depletion	
6	cytoplasmic alteration eosinophilic	17
7	cytoplasmic alteration glycogen accumulation	4
8	degeneration hydropic	2
9	fibrosis	2
10	focus of cellular alteration basophilic	1
11	focus of cellular alteration clear cell	1
12	hepatocellular adenoma	1
13	hepatocellular atrophy	1
14	hypertrophy and vacuolation	1
15	hypertrophy hepatocellular	48
16	hypertrophyhyperplasia	9
17	inclusions cytoplasmic	2
18	increased mitoses	4
19	karyocytomegaly	1
20	necrosis	1
21	necrosis zonal	2
22	oval cell hyperplasia	1
23	pigmentation pigment deposition	3
24	prominent nucleoli	1
25	single cell necrosis	10
26	vacuolation	19
27	vacuolation lipid	3

## Supporting Information Available

The INHAND annotation for TG-GATEs (XLSX)
